# Epidemiological and clinical features of primary biliary cholangitis in two Croatian regions: **a** retrospective study

**DOI:** 10.3325/cmj.2019.60.494

**Published:** 2019-12

**Authors:** Anita Madir, Tonći Božin, Ivana Mikolašević, Sandra Milić, Davor Štimac, Maja Mijić, Tajana Filipec Kanižaj, Zrinka Biloglav, Marko Lucijanić, Iva Lucijanić, Ivica Grgurević

**Affiliations:** 1 Department of Gastroenterology, Hepatology and Clinical Nutrition, University Hospital Dubrava, Zagreb, Croatia; 2 University of Zagreb School of Medicine, Zagreb, Croatia; 3 Department of Gastroenterology and Hepatology, University Hospital Centre Rijeka, Rijeka, Croatia; 4 School of Medicine, University of Rijeka, Rijeka, Croatia; 5 Department of Gastroenterology and Hepatology, University Hospital Merkur, Zagreb, Croatia; 6 Department of Medical Statistics, Epidemiology and Medical Informatics, Andrija Štampar School of Public Health, Zagreb, Croatia; 7 Department of Hematology, University Hospital Dubrava, Zagreb, Croatia; 8 Department of Dermatology and Venereology, County Hospital Karlovac, Karlovac, Croatia; 9 Faculty of Pharmacy and Biochemistry, University of Zagreb, Zagreb, Croatia

## Abstract

**Aim:**

To assess the measures of disease frequency and determine the clinical features of primary biliary cholangitis (PBC) in two Croatian regions.

**Methods:**

Databases of two tertiary hospitals, one located in the continental and one in the coastal region of Croatia, were retrospectively searched for PBC patients diagnosed from 2007 to 2018. Epidemiologic data analysis was restricted to patients from each hospital’s catchment area. We analyzed factors related to response to therapy and event-free survival (EFS), defined as absence of ascites, variceal bleeding, encephalopathy, hepatocellular carcinoma, liver transplantation (LT), or death. In addition, we determined clinical and demographic data of transplanted PBC patients.

**Results:**

Out of 83 PBC patients, 86.7% were female, with a median age at diagnosis of 55 years. Average PBC incidence for the 11-year period was 0.79 and 0.89 per 100 000 population, whereas the point prevalence on December 31, 2017 was 11.5 and 12.5 in the continental and coastal region, respectively. Of 76 patients with complete medical records, 21% had an advanced disease stage, 31.6% had an associated autoimmune condition, and all received ursodeoxycholic acid. EFS rate at 5 years was 95.8%. In an age and sex-adjusted multivariate Cox regression model, the only factor significantly associated with inferior EFS was no response to therapy (HR = 18.4; *P* = 0.018). Of all Croatian patients who underwent LT, 3.8% had PBC, with the survival rate at 5 years after LT of 93.4%.

**Conclusion:**

This study gives pioneer insights into the epidemiological and clinical data on PBC in Croatia, thus complementing the PBC map of Southeast Europe.

Primary biliary cholangitis (PBC) is a chronic cholestatic liver disease, an autoimmune disease activated by still unknown environmental factors in genetically susceptible individuals (1-3). The disease primarily affects cholangiocytes, leading to ductal destruction and loss. Its diagnostic hallmark are antimitochondrial antibodies (AMA), which are directed against the E2 subunit of the pyruvate dehydrogenase complex in combination with chronic cholestasis, whereas liver biopsy is reserved only for serologically negative patients and patients suspected of having an overlap with other diseases, such as autoimmune hepatitis or sclerosing cholangitis (PSC) (4,5). Common clinical presentations include fatigue, right upper abdominal pain, and pruritus, accompanied by a range of different other autoimmune phenomena, while 50% of patients are asymptomatic (5-7). The worldwide incidence and prevalence rates per 100 000 population range from 0.33 to 5.8 and from 1.91 to 40.2, respectively (8). The highest prevalence was reported in North America and Northern Europe, namely Great Britain and the Scandinavian countries (9), suggesting a west-east and north-south declining gradient (8). Most of the data about European population come from studies in Western European countries, while there are no population-based epidemiological studies in Central and South-East Europe. In Croatia, no data on PBC incidence and prevalence rates have been published. This makes it urgent to fill the knowledge gap about the disease frequency and identify and more precisely quantify the risk factors and potential environmental triggers. The aims of this study were 1) to gain preliminary insights into PBC incidence and prevalence, based on hospital database analysis from two centers located in different geographic regions of Croatia; 2) to determine the clinical features and outcomes of PBC patients from these two centers, and 3) to determine the clinical features and outcomes of PBC patients who underwent liver transplantation (LT).

## PATIENTS AND METHODS

### Study design and patient population

This retrospective hospital-based study was conducted from 2007 till 2017 at two tertiary hospitals: University Hospital Dubrava, Zagreb (UHD), located in northwestern Croatia, and University Hospital Center Rijeka (UHR), located in southwestern Croatia. According to the organization of the national health service and 2011 Census data, these hospitals provide health care services to the catchment areas of 331 288 and 296 195 inhabitants, respectively. Each patient’s residence in the hospital’s catchment area was verified by checking the records in the hospital’s electronic database. We also analyzed clinical characteristics, response to ursodeoxycholic acid (UDCA) treatment, survival, and the occurrence of liver related complications based on available follow-up data. Furthermore, we analyzed and descriptively presented clinical characteristics of the patients who underwent LT at the University Hospital Merkur (UHM), Zagreb, from 2008 to 2019. The UHM is the highest-volume LT center in Croatia, performing 115-130 LTs per year. Patients from this center were not included into the epidemiological analyses since they originated from all over Croatia and were selected according to the severity of liver disease and the need for LT.

### Case-finding method and diagnostic criteria

Three trained medical doctors searched electronic databases of the UHD and UHR for the records on all patients diagnosed with PBC according to the current EASL guidelines (5) from January 1, 2007 till December 31, 2017, and the database of UHM for patients diagnosed from January 1, 2008 till December 31, 2018. Patients presenting with persistent cholestatic liver test abnormalities, including elevated alkaline phosphatase (ALP) and/or gamma-glutamyltransferase (GGT) levels, with or without hyperbilirubinemia, were screened for the presence of AMA. Patients positive for AMA were diagnosed with PBC, while others required liver biopsy to confirm the diagnosis.

### Study outcomes

PBC incidence was estimated for the population residing at the catchment area of UHD and UHR for the period 2007-2018. Incidence was calculated as the number of new PBC cases within one year in the numerator and the size of the catchment population in the denominator. Point prevalence on December 31, 2017 was calculated as the number of people with PBC in the numerator and the size of the catchment population in the denominator. To assess the prevalence, we identified all PBC patients alive at this date residing in the catchment areas of each of the two centers (including those who were diagnosed before 2007: from 1996 at UHD and from 1998 at UHR). Both measures of disease frequency were expressed per 100 000 general population per year (10).

We also obtained data on the clinical features of PBC patients: age of disease onset, sex, liver function tests, AMA status, and the presence of other autoimmune diseases. Patients were further categorized as having early stage PBC, defined by histologic grade I-II or by normal bilirubin and albumin values at enrolment in patients without liver biopsy, or advanced-stage disease (11). Response to UDCA therapy was evaluated in the subsample of patients who had available laboratory data across a 12-month period according to Paris II criteria (11). Non-adherence to therapy was self-reported by patients at regular visits. When follow-up data were available, we analyzed survival and the occurrence of liver-related complications. Event free survival (EFS) was determined considering a composite endpoint defined as the absence of either liver decompensation (ascites, variceal bleeding, hepatic encephalopathy, and/or jaundice), hepatocellular carcinoma (HCC), death of any cause, and/or LT, whichever occurred first. For PBC patients who underwent transplantation, we assessed pre-transplant characteristics and conducted LT outcome analysis. This study was approved by the Ethics Committees of University Hospital Dubrava (2019/0602-04), University Hospital Center Rijeka (2170-29-02/1-19-2), and University Hospital Merkur (03/1-4280/2), and all procedures were in accordance with the Helsinki Declaration of 1975, as revised in 2008.

### Statistical analysis

Normality of distribution was tested using the Shapiro-Wilk test. Normally distributed numerical variables are presented as mean ± standard deviation (SD) and were compared between the groups with use of the *t* test. Non-normally distributed numerical variables are presented as median and interquartile range (IQR) and were compared between the groups with use of the Mann-Whitney U test. Categorical variables are presented as ratio and percentage and were compared between the groups with use of the Fisher exact test or χ^2^ test, where appropriate. Multivariate assessment of factors related to response to therapy was done with use of the logistic regression. Survival analyses were based on Kaplan-Meier method. Survival curves were compared using the Cox-Mantel version of the log-rank test (12). Data were screened for significant associations with survival using a custom made MS Excel workbook (13). Multivariate assessment of factors related to the time to event of interest was done using the Cox regression. The level of significance was set at *P* < 0.05. Analyses were performed with the MedCalc Statistical Software, version 18.5 (MedCalc Software bvba, Ostend, Belgium).

## RESULTS

In total, 123 PBC patients were identified: 47 at UHD, 36 at UHR, and 40 at UHM. Of these, 74 patients resided in the catchment areas of UHD and UHR and were eligible for the analysis of PBC prevalence, 76 patients with retrievable data sets at the time of diagnosis were eligible for the assessment of clinical characteristics, 62 patients with available follow-up data were eligible for the assessment of response to UDCA treatment, and 40 transplanted patients were eligible for the assessment of pre-transplant characteristics of PBC LT candidates and LT outcome analysis.

### Epidemiological analysis

**Figure 1:**
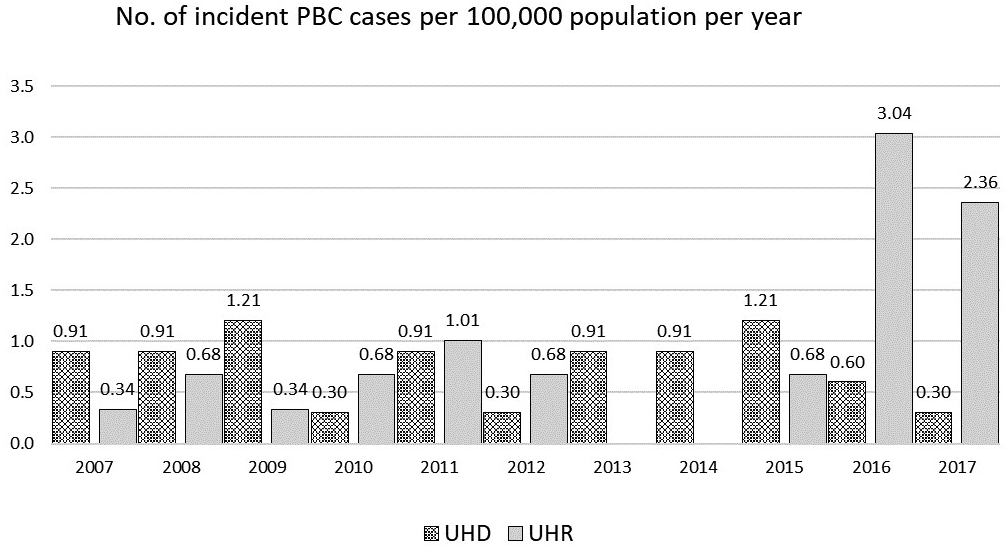
The number of incident cases of primary biliary cholangitis (PBC) per 100 000 population per year diagnosed at two Croatian tertiary hospitals from 2007 to 2018. Checkered – University Hospital Dubrava (UHD); gray – University Hospital Rijeka (UHR).

When the epidemiological analysis was restricted to the patients residing in the catchment area of UHD (N = 38) and UHR (N = 36) who were alive on December 31, 2017, the point prevalence was 11.5 and 12.5 per 100 000 population, respectively. The annual incidence per 100 000 population for the period from 2007 to 2018 ranged from 0.3 to 1.21 in the catchment area of UHD (average incidence 0.79) and from 0.34 to 3.04 in the catchment area of UHR (average incidence 0.89) (Figure 1). If these rates were representative of Croatian population, the estimated number of PBC patients on December 31, 2017 would be between 492 and 535.

### Clinical features of non-transplanted PBC patients

Out of 83 PBC patients from both centers, 72 (86.7%) were female, with the median age of 55 years at diagnosis (IQR 51-63; range 16-76). Seventy-six patients had all relevant clinical data available (Table 1). Among these 76 patients, 11 (14.5%) had overlap syndromes with other autoimmune liver diseases: 9/76 (11.8%) with autoimmune hepatitis and 2/76 (2.6%) with PSC. Other autoimmune diseases were present in 24/76 (31.6%) patients, namely rheumatoid arthritis in 10/76 (13.2%), Hashimoto thyroiditis in 10/76 (13.2%), and systemic scleroderma in 4/76 (5.3%) patients. Sicca syndrome, Sjogren syndrome, Graves’ disease, and celiac disease were present in one patient each.

**Table 1:** Patients characteristics for the whole cohort and stratified by response to ursodeoxycholic acid (UDCA)

Characteristics	Whole cohort (n = 76)	No response to treatment (n = 8)	Response to treatment (n = 54)	*P*
Age (years), median (IQR)	56 (51-63)	56 (51-62)	63 (53-67)	0.141
Male sex, n (%)	9/76 (11.8)	0/8 (0)	8/54 (14.8)	0.581
AMA positive, n (%)	56/76 (73.7)	7/8 (87.5)	39/54 (72.2)	0.668
AMA M2 positive, n (%)	31/58 (53.4)	4/5 (80)	26/48 (54.2)	0.374
ANA positive, n (%)	25/65 (38.5)	4/7 (57.1)	19/49 (38.8)	0.429
IgM (g/L), median (IQR)	3.1 (1.9-4.5)	4.4 (4.4-5.1)	3 (2-4.5)	0.089
Nausea, n (%)	23/76 (30.3)	3/8 (37.5)	12/54 (22.2)	0.388
Fatigue, n (%)	27/76 (35.5)	1/8 (12.5)	20/54 (37)	0.247
Pruritus, n (%)	18/76 (23.7)	3/8 (37.5)	13/54 (24.1)	0.414
Osteoporosis, n (%)	16/76 (21.1)	2/8 (25)	12/54 (22.2)	1.000
Comorbid autoimmune diseases, n (%)	24/76 (31.6)	3/8 (37.5)	17/54 (31.5)	0.705
Advanced stage, n (%)	13/62 (21)	5/8 (62.5)	8/54 (14.8)	0.008
Histological stage, n (%)				
0	1/33 (3)	0/2 (0)	1/31 (3.2)	0.019
1	9/33 (27.3)	0/2 (0)	9/31 (29)	
2	15/33 (45.5)	0/2 (0)	15/31 (48.4)	
3	4/33 (12.1)	1/2 (50)	3/31 (9.7)	
4	3/33 (9.1)	1/2 (50)	2/31 (6.5)	
Overlap syndrome, n (%)	11/76 (14.5)	1/8 (12.5)	10/54 (18.5)	1.000
Initial UDCA dose (mg), median (IQR)	1000 (750-1500)	1000 (1000-1000)	1000 (750-1500)	0.697
Dose reduction, n (%)	8/60 (13.3)	0/6 (0)	8/54 (14.8)	0.585
Corticosteroids, n (%)	15/62 (24.2)	3/8 (37.5)	12/54 (22.2)	0.388
Other immunosuppressive drug, n (%)	5/62 (8.1)	1/8 (12.5)	4/54 (7.4)	0.511
Baseline bilirubin (μmol/L), median (IQR)	11 (8.2-18)	11.3 (7.6-21.5)	11 (8.2-16)	0.764
Baseline ALT (U/L), median (IQR)	43.5 (34-77.5)	46 (23-57.5)	43.5 (34-79.8)	0.266
Baseline AST (U/L), median (IQR)	42.5 (31-65.5)	35 (22-76.3)	43 (32-61)	0.389
Baseline ALP (U/L), median (IQR)	189 (160-248)	283.5 (98.3-414.8)	188 (163-245)	0.593
Baseline GGT (U/L), median (IQR)	123.5 (87.3-344.8)	91 (34-214.5)	126 (91.5-344.8)	0.193
Baseline cholesterol (mmol/L), mean (SD)	5.9 ± 1.4	5.9 ± 0.8	5.9 ± 1.4	0.974
Baseline triacylglycerols (mmol/L), median (IQR)	1.3 (1.1-1.5)	1.1 (1-1.3)	1.3 (1.1-1.5)	0.385
Baseline platelets ×10^9^/L, mean (SD)	254.5 ± 74.9	261.5 ± 64.9	253.4 ± 76.8	0.778
Baseline albumin (g/L), mean (SD)	41.2 ± 3.7	40.7 ± 3.1	41.3 ± 3.8	0.695
Bilirubin at 12 months (μmol/L), median (IQR)	11 (8-12.9)	9 (8.3-16.5)	11 (8-12.8)	0.841
ALT at 12 months (U/L), median (IQR)	30 (23.8-39)	32 (24-50)	30 (24-39)	0.738
AST at 12 months (U/L), median (IQR)	28 (23-39)	44 (26-55.5)	27 (23-34.8)	0.225
ALP at 12 months (U/L), median (IQR)	125 (89.8-151.3)	312 (155.5-346.5)	122 (90-142)	0.065
Platelets at 12 months ×10^9^/L, mean (SD)	217.3 ± 84.4	178.3 ± 94.5	221.9 ± 82.9	0.235
Albumin at 12 months (g/L), median (IQR)	42 (39-43)	40 (36.3-43.8)	42 (39.5-43)	0.824
Non-adherence to UDCA therapy, n (%)	3/61 (4.9)	3/8 (37.5)	0/53 (0)	0.002

*IQR – interquartile range; SD – standard deviation; AMA – antimitochondrial antibodies; ANA – antinuclear antibodies; ALT – alanine transaminase; AST – aspartate transaminase ; ALP – alkaline phosphatase; IgM – immunoglobulin M; GGT – gamma glutamyltransferase.

All patients received UDCA treatment with a median dose of 1000 mg (IQR 750-1500). The data from 62 patients were evaluated after 12 months, and 54/62 (87.1%) patients achieved response (Table 1). Factors univariately associated with a higher odds ratio (OR) of not achieving the response were advanced disease stage (*P* = 0.008), higher histological stage (*P* = 0.019), and non-adherence to therapy (*P* = 0.002). In an age- and sex-adjusted multivariate model, advanced stage of disease remained significantly associated with non-response to therapy with OR = 28.4 (*P* = 0.006). Histological stage was omitted from the analysis due to incomplete data.

**Figure 2:**
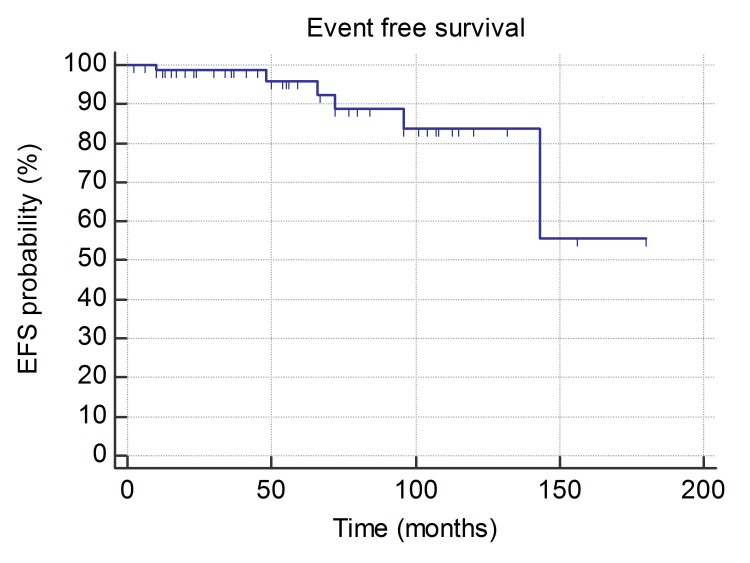
Event free survival (EFS) curve of primary biliary cholangitis (PBC) patients

The median follow-up of our patients lasted 39 months. During the follow-up, one patient died due to a liver-unrelated cause and five patients experienced liver decompensation. Since only one death was recorded in the whole group of patients, no further multivariate analyses regarding overall survival were performed. EFS rate at 5 years was 95.8%, and median EFS time was not reached (Figure 2). Factors univariately associated with shorter EFS were age at diagnosis >59 years (hazard ratio [HR] = 4.7; *P* = 0.038), osteoporosis (HR = 4.8; *P* = 0.043), advanced disease stage (HR = 16.9; *P* = 0.001), ALP≤110 (HR = 8; *P* = 0.031), total cholesterol ≤4.7 (HR = 7.7; *P* = 0.011), triacylglycerols (TAG)≤1.22 (HR = 11.8; *P* = 0.038), platelets ≤188 (HR = 13.4; *P* = 0.002), albumin <39 (HR = 9; *P* = 0.005), corticosteroid therapy (HR 5.8; *P* = 0.038), non-adherence to therapy (HR = 9.4; *P* = 0.017), and no response to therapy at 12 months (HR = 12; *P* = 0.001). These parameters were analyzed in a series of age- and sex-adjusted multivariate Cox regression models. The factors that remained significant after adjustments were total cholesterol ≤4.7 (HR = 14.4; *P* = 0.047), platelets ≤188 (HR = 48.2; *P* = 0.022), albumin <39 (HR = 39.8; *P* = 0.032), and no response to therapy (HR = 16.8; *P* = 0.024). When we analyzed these factors together in an age- and sex-adjusted multivariate Cox regression model, the only factor significantly associated with inferior EFS remained no response to therapy (HR = 18.4; *P* = 0.018).

### Clinical features of LT candidates with PBC

Among 1051 patients who underwent LT at the UHM in the period 2008–2019, 40 (3.8%) were transplanted due to PBC. Of them, 97.5% were women, and median age at LT was 59 years (IQR 53-63). In all patients, the primary indication for LT was liver cirrhosis with complications, while 2/40 (5%) also had HCC, 2/40 (5%) had overlap with PSC, and 1/40 (2.5%) had intractable pruritus.

Median model of end-stage liver disease score was 17 (IQR 14-21). Overall survival at 5 years was 93.4%. Two (2/40, 5%) patients died due to cardiovascular complications more than 1 year after LT. Four patients (4/40, 10%) experienced biopsy-proven disease relapse at a median of 4.5 years (IQR 4-5). All relapses at diagnosis occurred in early disease stages, except in one patient, with a relapse 9 years after LT, who was in advanced stage of fibrosis. Following the confirmed relapse of PBC, all patients were treated with UDCA. None of the patients died or were re-transplanted due to a relapse of PBC.

## DISCUSSION

In our study, the incidence of PBC per 100 000 population per year ranged between 0.3 and 1.21 in UHD and between 0.34 and 3.04 in UHR catchment area. For the respective geographical regions, the point prevalence on December 31, 2017 was 11.5 and 12.5 per 100 000 population.

Primary biliary cholangitis fits well to the definition of a rare disease (14). Epidemiological data are scarce and mainly collected in the western countries, with a few exemptions from the Eastern hemisphere (8). To put our data into the regional context, PBC incidence and prevalence in the Czech Republic were 3.0 and 11.5 per 100 000 inhabitants, respectively, and in Slovakia 1.45 and 14.5 per 100 000 inhabitants, (courtesy of Prof. Lubomir Skladany, Banska Bystrica, Slovakia, personal communication). In the Italian population in Lombardy, the incidence was 1.67/100 000 and the prevalence was 16 per 100 000 (15). These results support the existence of a declining northwest and southeast epidemiological gradient within the European region. Still, the Greek island of Crete should be considered as an outlier, with the incidence of 2.1 per 100 000 and prevalence of 36.5 per 100 000 (16). The disparities in geographical distribution could be explained by different genetic background and environmental factors (8,16).

The temporal incidence trends observed in our study should be interpreted with caution since our sample size is rather small. However, despite limitations, the suspected rising incidence is in line with the global trends. The worldwide increase in the PBC prevalence could be facilitated by incidence rates in female population (8,17). The trends observed in our study can be explained by the improvements in diagnostic tools, increased disease awareness, easier access to patients’ data as a result of digitalized patient registration, and potentially improved survival upon UDCA treatment (although the data on the latter are equivocal) (18-23).

As much as 86.7% patients in our study were middle-aged women and 73.7% were AMA positive. Symptoms were present in 50% of patients, most prevalently fatigue, nausea, pruritus, and osteoporosis. Liver overlap syndrome coexisted in 14.5% and other autoimmune diseases in 31.6% of patients, respectively. The clinical characteristics of our PBC patients were similar to those described previously (2).

We also assessed patients’ EFS and response to UDCA therapy, as well as the factors related to both. Since only one patient in the entire cohort died, survival could not be further analyzed. However, EFS was excellent, reaching 95.8% at 5 years of follow-up, most probably because the majority of patients had earlier clinical stages of PBC at diagnosis. In these patients, UDCA therapy is expected to be more effective, leading to better survival. Indeed, the only factor that remained independently associated with a better outcome, after adjustments for other factors in the multivariate Cox regression analysis, was response to UDCA therapy. On the other hand, the factor that was independently associated with a good response to UDCA therapy (according to Paris II criteria) was early-stage liver disease. Early clinical stage of PBC and adherence to UDCA therapy in the majority of patients also explain such favorable EFS in our study. UDCA at a daily dose of 13-15 mg/kg represents the mainstay of therapy, with a 50%-75% success rate (5). Therapy improves serum liver tests and slows down the rate of histologic progression, without having consistent effects on the symptoms (24-26). This is mostly true for patients in the early stage of PBC, although other studies reported controversial data (26,27). Several risk factors for disease progression and poor response to therapy were identified: female sex, younger age, symptom presence, baseline albumin and bilirubin values, liver stiffness values measured by transient elastography >9.6 kPa, and advanced histologic stage (28-32). For patients with poor response to therapy, obeticholic acid has been recently licensed a second-line option, acting as a powerful farnesoid receptor agonist with promising results (33).

In the transplanted cohort, 97.5% patients were female, pointing to a possible negative impact of female sex on the disease course. All patients were transplanted due to complications of liver cirrhosis. Interestingly, the median age at LT was only four years higher than the age in the non-transplanted group. This observation might be explained by the fact that transplanted patients have unidentified factors responsible for a faster development of cirrhosis coupled with unresponsiveness to UDCA treatment at advanced disease stages. However, we could not determine the exact effect of no response to UDCA and the exact disease stage at diagnosis due to the retrospective nature of study and the referral of the patients to the LT center only at the terminal stage of liver disease. Even though graft survival in our patients was 100%, biopsy-proven recurrent PBC at a median time of 4.5 years reached 10%. This was in accordance with published data, which show that the recurrence of autoimmune diseases, including PBC, at a median time of 5 years ranges between 10% and 50%; the differences may be partly attributable to the use of protocol vs clinically indicated liver biopsies and different diagnostic criteria in LT centers (34,35). However, the recurrent disease progresses slowly and has a minimal impact on graft function and patient survival (36).

The presented results could be partly influenced by study limitations. This study was limited to the tertiary care hospitals’ databases since other data sources, such as pathology archives or public health registers, are not established for PBC patients. Studies of PBC epidemiology have used various sources for case-finding, but the search of medical record databases, despite its limitations, was the most common approach (8). Although the hospital catchment areas were precisely geographically defined, it was not possible to determine the exact number of patients within the study base. This can be partly attributable to the lack of symptoms or nonspecific symptoms among PBC patients. Some patients might not have been recognized and referred to specialist care in the hospital, possibly leading to underdiagnosis, as previously described (8). It is a common popular belief that patients with advanced liver disease are addicted to alcohol (and in some cases there is overlap indeed), further leading to underestimation, as previously reported (8). Data obtained by a retrospective review of medical records may be incomplete or missing. It was difficult to retrieve the data for patients diagnosed before 2007, when the electronic database was established, and some important data were missing, so we limited our study to the period from 2007 till 2018. The reliance on diagnoses recorded in electronic medical records also makes it difficult to standardize the diagnostic criteria, which is why we manually reviewed all cases and excluded questionable diagnoses, ensuring standardization to the greatest extent possible. Also, some patients lacked histologic data as the diagnosis was established by an alternative approach, such as specific AMA antibody testing. However, we believe that these numbers could not be significantly higher since most of the patients would have been registered during the follow-up at their regular visits. Additional limitations of our study are retrospective design, rarity of the disease (14), and low number of events during the follow-up period, affecting the statistical power of the presented findings.

In conclusion, this is a pioneer work on epidemiology of PBC in Croatia. Despite limitations, it gives new insight into the occurrence patterns of PBC in Europe, but further research is needed to fill the gap in data availability and verify our findings.
